# Antioxidant Properties of Polyphenolic Extracts from *Quercus Laurina*, *Quercus Crassifolia*, and *Quercus Scytophylla* Bark

**DOI:** 10.3390/antiox7070081

**Published:** 2018-06-26

**Authors:** Eréndira Valencia-Avilés, Martha Estrella García-Pérez, Ma. Guadalupe Garnica-Romo, Juan de Dios Figueroa-Cárdenas, Esperanza Meléndez-Herrera, Rafael Salgado-Garciglia, Héctor E. Martínez-Flores

**Affiliations:** 1 Programa Institucional de Doctorado en Ciencias Biológicas, Universidad Michoacana de San Nicolás Hidalgo, Ciudad Universitaria, Morelia, Michoacán CP 58030, Mexico; evalencia@umich.mx; 2 Facultad de Químico Farmacobiología, Universidad Michoacana de San Nicolás de Hidalgo, Tzintzuntzan 173, Col. Matamoros, Morelia, Michoacán CP 58240, Mexico; margarc@live.ca; 3 Facultad de Ingeniería Civil, Universidad Michoacana de San Nicolás de Hidalgo, Ciudad Universitaria, Morelia, Michoacán CP 58030, Mexico; ggarnica@umich.mx; 4 Centro de Investigación y Estudios Avanzados del Instituto Politécnico Nacional, Libramiento Norponiente 2000, Fraccionamiento Realde Juriquilla, Querétaro, Qro CP 76230, Mexico; jfigueroa@cinvestav.mx; 5 Instituto de Investigaciones sobre Recursos Naturales, Universidad Michoacana de San Nicolás de Hidalgo, Avenida San Juanito Itzícuaro SN, Morelia, Michoacán CP 58330, Mexico; emelendez@umich.mx; 6 Instituto de Investigaciones Químico Biológicas, Universidad Michoacana de San Nicolás de Hidalgo, Ciudad Universitaria, Morelia, Michoacán CP 58030, Mexico; rsalgado@umich.mx

**Keywords:** *Quercus*, oak bark, scavenging ability, polyphenols

## Abstract

The objective of this work was to determine the concentration of total phenols, total flavonoids, hydroxycinnamic acids, and proanthocyanidins present in crude extracts of *Quercus laurina*, *Q. crassifolia*, and *Q. scytophylla* bark. They were extracted by ethanol (90%) maceration and hot water. The antioxidant capacity was determined by the ability to capture OH•, O_2_•^−^, ROO•, H_2_O_2_, NO•, and HClO. The hot water crude extract of *Q. crassifolia* was chosen to be concentrated and purified due to its higher extraction yield (20.04%), concentration of phenol compounds (747 mg gallic acid equivalent (GAE)/g, 25.4 mg quercetin equivalent (QE)/g, 235 mg ChAE/g, 25.7 mg chlorogenic acid equivalents (ChAE)/g), and antioxidant capacity (expressed as half maximal effective concentration (EC_50_, µg/mL): OH• = 918, O2•^−^ = 80.5, ROO• = 577, H_2_O_2_ = 597, NO• ≥ 4000, HClO = 740). In a second stage, *Q. crassifolia* extracted with hot water was treated with ethyl acetate, concentrating the phenol compounds (860 mg GAE/g, 43.6 mg QE/g, 362 ChAE/g, 9.4 cyanidin chloride equivalents (CChE)/g) and improving the scavenging capacity (OH• = 467, O2•^−^ = 58.1, ROO• = 716, H_2_O_2_ = 22.0, NO• ≥ 4000, HClO = 108). *Q. crassifolia* had the highest polyphenolic concentration and the better capacity for scavenging reactive species, being a favorable candidate to be considered in the development of new products.

## 1. Introduction

The genus *Quercus*, common name oak, belongs to the Fagaceae family. Oak is one of the most profitable economic woods used in the cooperage industry [1]; even the bark is utilized as stoppers. However, up to 25% of the bark has no application or is consumed as fuel [2]. The bark is then considered as waste, with scarce utilization, and sometimes the improvised ways of storage, elimination, or distribution can become an ecological and economic problem to be solved [3].

Some studies [4,5,6], reported that oak bark contains high concentrations of polyphenols. Structurally, phenolic compounds comprise an aromatic ring, bearing one or more hydroxyl substituents, and range from simple molecules to highly complex polymerized compounds [7]. More than 8000 phenolic structures are currently known, including phenolic acids (derivatives of benzoic acid and cinnamic acid), flavonoids, tannins (hydrolyzable and condensed), stilbenes, and lignans, among others [8]. In plant tissues, they are mainly present attached to sugars, although occasionally they are found as aglycones [9].

Since polyphenols are capable of scavenging reactive species, such as superoxide anions (O_2_•^−^), hydroxyl (•OH), nitric oxide (NO•), and alkyl peroxyl radicals (ROO•), they are linked to the prevention of various diseases, by acting as antioxidants through electron-donating mechanisms [10]. Polyphenols have different types of pharmacological properties, including antioxidant, anti-inflammatory, antiproliferative, and hypolipidemic, and they also regulate redox potentials, metabolic disorders, and apoptosis [11].

Most research about the phenolic content and antioxidant properties of oak has been done on European and Asiatic species, mainly using leaves [12], twigs [13], acorns [14], and bark [15], and has shown that oak bark contains the highest concentration of total phenols, including tannins, proanthocyanidins, and flavonoids with strong anti-radical properties [4,5,15].

Although Mexico is considered one of the most important countries regarding the diversity of oaks, with 150 species reported [16], and the traditional use of the bark in Mexican ethnic groups has been to treat different illnesses, like stomach ache, burns, infections, pain, etc. [17], the scientific information about Mexican oak species is scarce. Bark extract from *Q. sideroxyla* was determined to have a high concentration of total phenols, proanthocyanidins, and flavonoids, as well as strong antioxidant activity [3]. The chemical composition of sapwood, heartwood, and bark of the Mexican species *Q. laurina* and *Q. crassifolia*, was described, concluding that bark extract content for *Q. crassifolia* and *Q. laurina* was higher in comparison to other hardwood bark [6]. 

Bark oak polyphenols have been used for the development of functional foods, such as, the use of oak wood during the elaboration of barrels in the aging winemaking process, that contributes to increasing the polyphenol content of wines, due to the extraction of these compounds from oak wood [18]. Moreover, the preparation of drinks based on toasted acorn [19], honeydew honey of *Q. pyrenaica* [20], and infusions of oak leaves [21], show that oak is a good source of phenolic compounds, which can be extracted and used in the formulation of new functional products.

Oak bark from Mexican species represents an area of great interest for research, because it is a potential source of natural antioxidants [22]. The objective of this study was to determine the presence and amount of different groups of polyphenols in extracts from oak species *Quercus crassifolia*, *Quercus laurina*, and *Quercus scytophylla*, as well as their antioxidant capacity. We used two extraction methods, maceration with ethanol solution (90%) and extraction with hot water. Also, we selected the oak (*Q. crassifolia*), which showed both the highest polyphenol yield and highest antioxidant capacity in the extracts to be purified with ethyl acetate, to obtain an extract rich in polyphenol compounds. 

## 2. Materials and Methods

### 2.1. Raw Material

Bark of *Q. crassifolia*, *Q. laurina*, and *Q. scytophylla* was collected in a forestry plantation located in Ciudad Hidalgo, Michoacán, México. The three oak species were chosen due to their importance for the forestry industry in Michoacán. The leaves, flowers, and acorn were botanically identified by Pablo Cuevas Reyes, expert in forest botany of the Biology Faculty, Universidad Michoacana de San Nicolás de Hidalgo (UMSNH), and Emmanuel Pérez Calix, botanist of the “Institute of Ecology” in Patzcuaro, Michoacán. The bark was washed, cut into rectangles of about 5 × 5 cm, and dried at 40 °C for 48 h. The bark was milled using a Thomas Digital ED-5 Wiley^®^ cutting mill and sieved with # 40 mesh (400 µm). 

### 2.2. Polyphenol Crude Extracts

#### 2.2.1. Extraction by Maceration

The crude extraction of phenolic compounds was done by maceration [23]. Twenty grams of powder bark was added to 200 mL of a 90% ethanol solution, and agitated at 220 rpm for 24 h at 22 °C. The extract was filtered using Whatman® 42 paper, and the solids were washed with 200 mL of 90% ethanol. The crude extract was evaporated and then the solid residue was lyophilized (FreeZone 2.5 Liter Benchtop Freeze Dry System, Labconco®, Kansas City, MO, USA) and stored in amber bottles at 4 °C.

#### 2.2.2. Extraction by Hot Water

Fifty grams of powder bark was used for the extraction with water (2 × 500 mL) under reflux for 1 h. The aqueous extract was filtered with Whatman 42 filter paper, lyophilized, and stored in dark bottles at 4 °C [23].

### 2.3. Crude Extract Composition

Total phenol, flavonoid, hydroxycinnamic acid, and proanthocyanidin content were determined in the crude extracts using spectrophotometric techniques. The results were compared with the commercial extract Oligopin^®^ (Nutri-Dyn, Maple Plain, MN, USA), recognized for its antioxidant properties, as mentioned [24]. Total phenol content was determined following the method proposed by Scalbert et al. (1989), using a gallic acid calibration curve, and the absorbance was measured at 750 nm and the results were expressed as mg of gallic acid equivalent (GAE)/g of dried extract [25]. The flavonoid content was determined at 415 nm with the method of Brighente et al. (2007), using a calibration curve with quercetin used as standard. The results were expressed as mg of quercetin equivalent (QE)/g of dried extract [26]. The hydroxycinnamic acid content was determined by comparison with a calibration curve using chlorogenic acid as a standard, and the absorbance of the test was measured at 525 nm, according to the method described in the European Pharmacopoeia [27], while the results were expressed as mg of chlorogenic acid equivalents (ChAE)/g of dried extract. Proanthocyanidin content was determined at 550 nm, using cyanidin chloride as standard by the methodology proposed by Porter et al. (1996) [28]. The results were expressed as mg of cyanidin chloride equivalents (CChE)/g of dried extract.

### 2.4. Antioxidant Capacity of Crude Extracts

The antioxidant capacity of oak bark crude extracts was determined by spectrophotometric methods, considering the ability of extracts to capture oxidizing species of biological relevance. The percentage inhibition for hydrogen peroxide, nitric oxide, hypochlorous acid, superoxide, and hydroxyl was calculated using Equation (1), as reported by García-Pérez et al. (2010) [29]:

(1) $$\%\mathrm{Scavenging}=100\left[\frac{A_{0}-\left(A_{1}-A_{2}\right)}{A_{0}}\right],$$

where *A*_0_ is the absorbance of the mixture without the extract, *A*_1_ is the absorbance of the mixture with the extract, and *A*_2_ is the absorbance of the extract.

Equation (2) was used to determine the percentage of peroxyl radical scavenging [29]:

(2) $$\%\mathrm{Scavenging}=100\left[1-\frac{A_{e0}-A_{ef}}{A_{c0}-A_{cf}\;}\right],$$

where, *A_e_*_0_ is the absorbance of the mixture without 2,2-diazobis(2-amidi-nopropane) dihydrochloride (AAPH), *A_c_*_0_ is the control absorbance (absorbance of the mixture without extract or AAPH), *A_ef_* is the absorbance of the mixture with extract and AAPH, and *A_cf_* is the absorbance of the mixture without the extract.

In all cases, the effective concentration (EC_50_) was calculated, defined as the amount of extract required until reduced by 50% of the concentration of the reactive species. The antioxidant capacity of extracts was compared to that of Oligopin^®^ and turmeric Terana^®^ (Terana S.A., Ciudad de México, México) taken as positive controls. All analyses were done in triplicate.

#### 2.4.1. Superoxide Anion Radical (O_2_•^−^) Scavenging Activity

The ability of the extracts to capture superoxide radicals (O_2_•^−^) was measured [30], using one milliliter of nitroblue tetrazolium [100 µM] mixed with 1 mL of Nicotinamide adenine dinucleotide NADH [468 µM] and 1 mL of extract. The reaction was initiated with 150 µL of phenazine methosulfate. The solution was incubated at 30 °C for 30 min, and the absorbance of the solutions was measured [30].

#### 2.4.2. Hydrogen Peroxide (H_2_O_2_) Scavenging Activity

The capability of extracts to scavenge H_2_O_2_ reactive species was calculated following the procedure described by Ruch et al. (1989). In test tubes, 1.7 mL of the extract solution, in concentrations between 0.5 and 1500 µg/mL, plus 300 µL of H_2_O_2_ solution (40 mM) was added, and maintained at 22 °C for 3 min. The absorbance was measured at 230 nm [31].

#### 2.4.3. Hydroxyl Radical (OH•) Scavenging Activity

The antiradical activity of the extracts against hydroxyl radicals was determined as reported by Smirnoff & Cumbes (1989). One milliliter of the extract solution was added to dimethyl sulfoxide at different concentrations (ranging from 100 to 2000 µg/mL), 300 µL FeSO_4_ (8 mM), and 250 µL of H_2_O_2_ (20 mM). The reaction was initiated by addition of 1 mL of salicylic acid solution (3 mM), and then was incubated for 30 min at 30 °C. After that, 450 µL of distilled water was added, and the mixture was centrifuged for 10 min at 3500 rpm, the supernatant recovered and the absorbance measured at a wavelength of 510 nm [32].

#### 2.4.4. Nitric Oxide Radical (NO•) Scavenging Activity

The nitric oxide radical scavenging activity was analyzed according to the method indicated by Sreejayan & Rao (1997) where 0.5 mL aliquot of extract between 100 and 2500 µg/mL was mixed with 0.5 mL of 10 mM sodium nitroprusside, and placed in a water bath at 37 °C for 2.5 h. After that, the samples were maintained at 22 °C for 20 min, then 1 mL of Griess reagent was added; the mixture was kept for 40 min at 22 °C, and maintained for 20 min in the dark. The absorbance was measured at 548 nm [33].

#### 2.4.5. Peroxyl Radical (ROO•) Scavenging Activity

The ability to capture peroxyl radicals (ROO•) was determined by using the pyrogallol red (1.5 mL) as a target oxidized molecule and AAPH (25 µL, 600 mM) as a peroxyl radical generator like López-Alarcón & Lissi (2005). A 150 µL aliquot of extract was added at concentrations between 100 µg/mL and 1500 µg/mL. The oxidized pyrogallol red was measured at 540 nm [34].

#### 2.4.6. Hypochlorous Acid (HClO) Scavenging Activity

The capacity to scavenge hypochlorous acid was measured using the methodology described by Aruoma & Halliwell (1987) with some modifications. One milliliter of the extract, between 100 and 2500 µg/mL, was mixed with 1 mL of HClO (14.3 mM) in phosphate-buffered saline (PBS). The mixture was incubated at 37 °C for 15 min. Then, 222.22 µL of catalase (400 U) was added, and the mixture was incubated for 15 min at 37 °C. The absorbance was measured at 240 nm [35].

### 2.5. Liquid–Liquid Purification of the Most Antioxidant Extract Selected: Composition and Antioxidant Capacity of the Purified Extract

The crude extract of *Q. crassifolia* was chosen to be purified and concentrated, because it was shown to have the best extraction yield, higher polyphenol concentration, and better capacity to scavenge reactive species. A liquid–liquid extraction was done as follows [7]. Six grams of lyophilized extract was suspended in 100 mL of water and filtered with a Gooch crucible. The solution was defatted with hexane (5 × 100 mL), and then the polyphenols were concentrated with ethyl acetate (5 × 100 mL). This solvent was evaporated, resuspended in water and lyophilized. The percentage extraction as well as the total phenol, total flavonoid, hydroxycinnamic acid, and proanthocyanidin content was determined for the purified extract. Also, the antioxidant capacity of the extract to scavenge different reactive species (hydrogen peroxide, nitric oxide, hypochlorous acid, as well as hydroxyl, superoxide, and peroxyl radicals) was analyzed.

### 2.6. Statistical Analysis

The experimental results were expressed as mean ± standard error (SE) of three replicates. Results were analyzed by analysis of variance (ANOVA) (*p* < 0.05), and means separated by Duncan’s test. Spearman’s correlation test was used for correlations (*p* < 0.05). Student’s t-test was used to analyze differences in content of phenolic compounds and the antioxidant capacity of the crude and purified extract. Statistical analysis was done using STATISTICA 7.0 software (TIBCO Software Inc, Palo Alto, CA, USA) [36]. 

## 3. Results and Discussion

### 3.1. Yields and Chemical Composition of Crude Extracts from Quercus sp.

The extraction yields of the crude extracts obtained by the two extraction methods for the three different *Quercus* sp. are shown in Table 1. There were no significant differences (*p* < 0.05) between the extraction method using hot water compared to the maceration method for *Q. laurina* and *Q. scytophylla*. Extract from *Q. crassifolia* using hot water had the highest extraction yield, 20.0% *w/w* dry bark. These differences can be explained by the different polarity of the compounds present in the species of *Quercus* sp.

The total phenol, flavonoid, hydroxycinnamic acid, and proanthocyanidin content is shown in Table 2. The concentration of total phenols followed the order: *Q. laurina* exposed to the maceration method (756 mg GAE/g), *Q. crassifolia* exposed to the hot water method (746 mg GAE/g) and *Q. crassifolia* exposed to the maceration method (694 GAE/g). There were no significant differences (*p* < 0.05) between treatments, and values were similar to those obtained for the Oligopin^®^ sample (735 mg GAE/g). For total flavonoid content, extraction with hot water led to the highest total flavonoid content in *Q. crassifolia* (25.4 mg QE/g), four times higher than the Oligopin^®^ value (6.4 mg QE/g). The highest value for proanthocyanidin compounds was observed in the *Q. crassifolia* extract obtained by maceration (53.5 mg CChE/g), significantly different (*p* < 0.05) from the other treatments and lower than that obtained for Oligopin^®^ (69.2 mg CChE/g). The best extraction yields for hydroxycinnamic acids and proanthocyanidins were obtained when the maceration method was used. Our results are in agreement with those results described by García-Pérez et al. (2010), where the relationship between the extracts made both by maceration and with hot water was studied in different barks of Canadian wood species.

In agreement with those results described by García-Pérez et al. (2010) and Naima et al. (2015), in the first one, compared the relationship between maceration and hot water extraction for different barks of Canadian wood species. In another investigation, Naima et al. (2015), showed that the increase of the extract temperature improves the yield of polyphenols and hydrolyzable tannins extracted with hot water from *Acacia mollissima* bark [29,37].

The lower content of hydroxycinnamic acids and proanthocyanidins in Michoacán oak species compared to the commercial extract Oligopin^®^ could be due to analysis of the extracts in crude form, which must contain highly polar compounds (e.g., sugars), non-selectively extracted using these extraction methods, unlike the extract Oligopin^®^, which represents the ethyl acetate fraction of the aqueous extract of French maritime pine bark.

For this reason, we decided to purify the extract obtained by the hot water method for *Q. crassifolia*, the species with the highest solids yield. This purification was done with ethyl acetate. The results showed that the fraction purified with ethyl acetate had the highest values for total phenols (860 mg GAE/g), flavonoids (43.6 mg QE/g), and hydroxycinnamic acids (362 mg ChAE/g) compared to the other species (Table 2), regardless of the extraction method used. Even greater values were obtained than for the commercial extract Oligopin^®^. Only the proanthocyanidin value was lower in the purified *Q. crassifolia* ethyl acetate extract compared to all other species, regardless of the method used.

### 3.2. Antioxidant Capacity of Crude Extracts from Quercus sp.

The antioxidant capacity values of crude extracts of the oak species obtained by maceration and hot water, as well as the Oligopin^®^ and Terana^®^ turmeric, considering their ability to capture species of biological relevance, such as hydroxyl, superoxide and peroxyl radicals, hydrogen peroxide, nitric oxide, and hypochlorous acid, is shown in Table 3. 

This chemical characterization provides information on the chemical reactivity of the phenolic crude extracts for different free radicals and reactive species. The EC_50_ values showed that *Q. crassifolia* extracts, obtained both by maceration and hot water, had the best ability to capture superoxide anions (40.9 and 80.5 µg/mL, respectively), while *Q. crassifolia* extracted with hot water showed the best ability to capture hydroxyl radicals (918 µg/mL), better even than Oligopin^®^ (1271 µg/mL). *Q. scytophylla* extracted with hot water had the best ability to capture peroxyl radicals (390 µg/mL), followed by *Q. crassifolia* (577 µg/mL) and *Q. laurina* extracted with hot water (582 µg/mL), which had similar values to Oligopin^®^ (563 µg/mL).

However, *Q. scytophylla* extracts showed a lower ability to capture other reactive species compared to the other extracts. No differences in ability to capture H_2_O_2_ were found for *Q. crassifolia* extracted with hot water, *Q. crassifolia* by maceration, *Q. laurina* with hot water, and *Q. laurina* by maceration (*p* < 0.05).

The ability of the extracts to capture nitric oxide was evaluated at the same time with Terana^®^ turmeric extract (Terana S.A.) obtained from dried rhizomes of *Curcuma longa*, a material of golden color, used worldwide as a food additive. Some studies have been done in recent years related to the biological activity of curcumin, including its antioxidant, antifungal, antitumor, anti-inflammatory, and antibacterial properties, attributed to its high ability to capture nitric oxide [38]. The turmeric extract showed the best ability to capture nitric oxide (53.8 µg/mL), followed by *Q. laurina* extracted by maceration (149 µg/mL).

As for the ability to inhibit hypochlorous acid, *Q. laurina* extracted by maceration had the best antioxidant capacity (387 µg/mL), followed by *Q. crassifolia* (740 µg/mL), and *Q. laurina* extracted with hot water (774 µg/mL).

As we can see, the overall results highlighted that the extracts of *Q. crassifolia* obtained with hot water and *Q. laurina* by maceration had the highest efficiency to capture the different radicals; however, *Q. crassifolia* had a higher extraction yield (Table 1).

To identify association of the distinct kinds of polyphenolic content with antioxidant activity, a Spearman correlation analysis was performed (Table 4). The capacity to capture superoxide radicals was related to both hydroxycinnamic acid (*r* = −0.829, *p* = 0.001) and total phenolic (*r* = −0.567, *p* = 0.004) compounds. These results are in agreement with those reported by Natić et al. (2015), who found that the polyphenols present in mulberry fruits grown in Vojvodina possess high antioxidant–antiradical activity, acting as potent superoxide anion radical scavengers; the predominant phenolic acids were protocatechuic acid and ferulic acid, showing high positive correlation with their ability to scavenge superoxide anion radicals [39].

The flavonoid (*r* = −0.631, *p* = 0.001) and total phenol (*r* = −0.441, *p* = 0.035) content in the extracts correlated with the ability to capture hydroxyl radicals, as found in the *Matricaria pubescens* extracts [40]; their results revealed significant correlation between the phenolic and flavonoid compound content and antioxidant activity assessed by hydroxyl radical scavenging capacity.

The hydrogen peroxide radical scavenging activity was more strongly associated with total phenols (*r* = −0.821, *p* = 0.002) than hydroxycinnamic acids (*r* = −0.545, *p* = 0.007). The ability of Canadian wood species extracts to scavenge H_2_O_2_ was also associated with total phenol content [29].

The concentration of total flavonoids and the proanthocyanidin content correlated with their ability to capture hypochlorous acid, *r* = −0.532 (*p* = 0.009) and *r* = 0.687 (*p* = 0.0002), respectively. Also demonstrated, was a high association between flavonoid compounds and antioxidant activity in extracts from buckwheat hulls and flour [41]. In our study, total flavonoid concentration was correlated with the ability to capture nitric oxide (*r* = 0.511, *p* = 0.013). That means that a high concentration of both proanthocyanidins and total flavonoids shows a greater ability to capture hypochlorous acid and nitric oxide.

### 3.3. Chemical Composition and Antioxidant Capacity of Purified Q. crassifolia Extract

As previously mentioned, the *Q. crassifolia* extract obtained by the hot water method was, in turn, subjected to a subsequent extraction with ethyl acetate. This extract, from now on called purified, was analyzed to determine the content of its diverse groups of phenolic compounds and their antioxidant capacity, as shown in Table 2 and Table 3. The extraction yield of the purified *Q. crassifolia* decreased considerably compared to the extraction yield of crude extract of *Q. crassifolia* using the hot water method. This is reasonable because we concentrated the compounds, obtaining only the fraction corresponding to those compounds that are soluble in ethyl acetate.

A higher concentration of total phenols, total flavonoids and hydroxycinnamic acids was observed in the purified *Q. crassifolia* extract compared to crude extract of *Q. crassifolia* obtained using the hot water method (Table 2); however, the purified extract showed a lower proanthocyanidin content, consistent with the study done by Quettier-Deleu et al. (2000) [41], which also described a higher total phenol content and lower proanthocyanidin content in an ethyl acetate extract of *Picea mariana* bark compared to the aqueous fraction extract. This result can be explained if we consider that the aqueous fraction, which is discarded during the purification step, contains mainly polymeric proanthocyanidins, whereas the ethyl acetate fraction is mainly composed of oligomeric proanthocyanidins. Notably, it has been shown that oligomeric proanthocyanidins, due to their lower molecular weight, possess greater biological activity than polymeric compounds. In fact, these latter, because of their large size, are unable to cross biological membranes, and therefore, cannot interfere with the signaling pathways related to disease pathogenesis [42].

The purified extract had improved ability to capture hydroxyl radicals, superoxide anions, hydrogen peroxide, and hypochlorous acid, while for peroxyl radicals, the purified extract had an increased EC_50_ value compared to crude extracts. This may be due to loss in the aqueous fraction of compounds that may be related to the ability to capture peroxyl radicals, such as polymeric proanthocyanidins, hydrolyzable tannins and lignans, among others, which had not been determined in this work.

In general, the purified extract had a better antioxidant capacity compared to the aqueous extract of *Q. crassifolia*. With regard to nitric oxide radicals, neither crude nor purified extracts, at concentrations less than 4000 mg/mL, had ability to capture the nitrogen reactive species.

## 4. Conclusions

In this research, a high concentration of total phenols was found in the *Quercus* sp. bark crude extracts, including different classes of phenolic compounds, like flavonoids, hydroxycinnamic acids, and proanthocyanidins, confirming that the Mexican species oak barks analyzed in this work are a good source of phenolic compounds.

There were no differences in the content of total phenols or hydroxycinnamic acids between purified extract of *Q. crassifolia* and Oligopin^®^, and actually, for concentration of flavonoids, the purified extract of *Q. crassifolia* had a higher concentration. Regarding the antioxidant capacity, the purified extract showed, in general, better antioxidant characteristics than Oligopin^®^, being a good candidate to be considered in the development of new products with high antioxidant activity, however, further studies are necessary to determine the chemical composition of the extract, as well as the toxicological profile, in order to have a better understanding about its possible applications.

## Figures and Tables

**Table 1:** Extraction yields of crude and purified extracts from Mexican oak species obtained by hot water and maceration methods.

Extract	Extraction Method	% Extraction Yield (*w*/*w* Dry Bark) ^†^
*Quercus crassifolia*	Hot water	20.0 ± 7.7 ^a^ *
Purified *Quercus crassifolia*	Hot water	2.7 ± 0.3
*Quercus crassifolia*	Maceration	11.0 ± 1.0 ^b,c^
*Quercus laurina*	Hot water	14.2 ± 0.2 ^b^
*Quercus laurina*	Maceration	13.6 ± 0.1 ^b^
*Quercus scytophylla*	Hot water	6.8 ± 1.9 ^c,d^
*Quercus scytophylla*	Maceration	4.4 ± 0.2 ^d^

^†^ % (*w*/*w* dry bark). Means with different letters (^a^, ^b^, ^c^, ^d^) in the same column are significantly different at *p* < 0.05 (ANOVA, followed by Duncan’s test). Means with * in the same column are different at *p* < 0.05 (Student’s *t*-test, comparing crude and purified *Q. crassifolia* hot water extracts).

**Table 2:** Total phenol, total flavonoid, total hydroxycinnamic acid, and proanthocyanidin content of Mexican *Quercus* species extracts obtained by hot water and maceration methods.

Extract	Total Phenols (mg GAE/g)	Total Flavonoids (mg QE/g)	Hydroxycinnamic Acids (mg ChAE/g)	Proanthocyanidins (mg CChE/g)
*Q. crassifolia* hot water	747 ± 41 ^a^	25.4 ± 0.6 ^a^	235 ± 2 ^c^	25.7 ± 1.3 ^d^ *
Purified *Q. crassifolia* hot water	860 ± 6 *	43.6 ± 0.3 *	362 ± 13 *	9.4 ± 0.3
*Q. crassifolia* maceration	695 ± 62 ^a^	14.0 ± 0.3 ^d^	269 ± 37 ^b^	53.5 ± 1.0 ^b^
*Q. laurina* hot water	474 ± 44 ^b^	24.1 ± 1.1 ^b^	133 ± 4 ^e,f^	14.2 ± 0.4 ^e^
*Q. laurina* maceration	756 ± 17 ^a^	15.7 ± 0.2 ^e^	145 ± 17 ^e,f^	24.3 ± 1.8 ^d^
*Q. scytophylla* hot water	329 ± 38 ^c^	24.1 ± 0.5 ^b^	113 ± 3 ^e,f^	12.6 ± 2.3 ^e^
*Q. scytophylla* maceration	521 ± 40 ^b^	12.9 ± 0.3 ^c^	173 ± 13 ^d,e^	48.4 ± 3.8 ^c^
Oligopin^®^	736 ± 20 ^a^	6.4 ± 0.2 ^f^	337 ± 28 ^a^	69.2 ± 0.8 ^a^

Means with different letters (^a^, ^b^, ^c^, ^d^, ^e^, ^f^) in the same column are significantly different at *p* < 0.05 (ANOVA, followed by Duncan’s test). GAE, gallic acid equivalents; QE, quercetin equivalents; ChAE, chlorogenic acid equivalents; CChE, cyanidin chloride equivalents. Means with * in the same column are different at *p* < 0.05 (Student’s *t*-test, comparing crude and purified *Q. crassifolia* hot water extracts).

**Table 3:** Free radical scavenging of *Quercus* sp. bark hot water and ethanolic extracts.

Extract	OH• EC_50_ (µg/mL)	O_2_•^−^ EC_50_ (µg/mL)	ROO• EC_50_ (µg/mL)	H_2_O_2_ EC_50_ (µg/mL)	NO• EC_50_ (µg/mL)	HClO EC_50_ (µg/mL)
*Q. crassifolia* hot water	918 ± 9 ^c^ *	80.5 ± 0.7 ^e^ *	577 ± 40 ^c^	597 ± 162 ^b^ *	>4000 ^a^ *	740 ± 54 ^b^ *
Purified *Q. crassifolia* hot water	467 ± 50	58.1 ± 1.6	717 ± 9 *	22.0 ± 1.8	>4000 *	108 ± 25
*Q. crassifolia* maceration	2024 ± 198 ^b^	40.9 ± 16.4 ^e^	1747 ± 87 ^a^	653 ± 122 ^b^	873 ± 49 ^b^	1276 ± 40 ^a^
*Q. laurina* hot water	1257 ± 75 ^c^	629 ± 9 ^c^	582 ± 15 ^c^	727 ± 57 ^b^	>4000 ^a^	774 ± 192 ^b^
*Q. laurina* maceration	>4000 ^a^	3213 ± 917 ^b^	622 ± 48 ^c^	519 ± 116 ^b^	149 ± 17 ^d^	387 ± 86 ^c^
*Q. scytophylla* hot water	1865 ± 396 ^b^	> 4000 ^a^	390 ± 160 ^d^	1102 ± 49 ^a^	>4000 ^a^	866 ± 183 ^b^
*Q. scytophylla* maceration	>4000 ^a^	406 ± 135 ^d^	856 ± 24 ^b^	1050 ± 166 ^a^	661 ± 177 ^c^	953 ± 212 ^b^
Oligopin^®^	1271 ± 72 ^c^	104 ± 8 ^e^	563 ± 33 ^c^	174 ± 10 ^c^	>4000 ^a^	1310 ± 114 ^a^
Terana^®^ turmeric					53.8 ± 39.0 ^d^	

Means with different letters (^a^, ^b^, ^c^, ^d^, ^e^) in the same column are significantly different at *p* < 0.05 (ANOVA, followed by Duncan’s test). OH•; O_2_•^−^; ROO•; H_2_O_2_; NO•; HClO. Means with * in the same column are different at *p* < 0.05 (Student’s *t*-test, comparing crude and purified *Q. crassifolia* hot water extracts). EC_50_: half maximal effective concentration.

**Table 4:** Spearman coefficient for correlation between the scavenging capacity of the reactive species and the phenolic compound content.

	ROO•	O_2_•^−^	OH•	H_2_O_2_	NO•	HClO
**Total phenols**	0.298	−0.567 *	−0.441 *	−0.821 *	−0.070	−0.411
**Total flavonoids**	−0.005	−0.223	−0.631 *	−0.119	0.511 *	−0.532 *
**Total hydroxycinnamic acids**	0.403	−0.829 *	−0.545 *	−0.835 *	0.186	0.023
**Proanthocyanidins**	−0.261	−0.245	0.324	−0.056	−0.331	0.687 *

* Correlations are significant at *p* < 0.050.
